# [CO_2_] Alters Cyanobacterial Carboxysome Encapsulation and Redox State

**DOI:** 10.21203/rs.3.rs-4814625/v1

**Published:** 2026-02-19

**Authors:** Clair A. Huffine, Catherine G. Fontana, Rosanna L. Garris, Colin Sempeck, Jeffrey C. Cameron, Anton Avramov

**Affiliations:** 1 Department of Biochemistry, University of Colorado, Boulder, CO 80309, USA; 2 Renewable and Sustainable Energy Institute, University of Colorado, Boulder, CO 80309, USA; 3 Interdisciplinary Quantitative Biology Program (IQ Biology), BioFrontiers Institute, University of Colorado, Boulder, CO 80309, USA; 4 Department of Geological Sciences, Boulder, CO 80309, USA; 5 Molecular, Cellular, and Developmental Biology Department, Boulder, CO 80309, USA; 6 National Renewable Energy Laboratory, Golden, CO 80401, USA

**Keywords:** Carboxysome, Procarboxysome, Cyanobacteria, Redox, CO_2_ Modulation, roGFP, Climate Change, Photosynthesis

## Abstract

Responsible for fixing 25% of CO_2_ globally, cyanobacteria use carboxysomes to house their CO_2_ fixing machinery. The formation and permeability of the proteinaceous shell of carboxysomes is an area of active study. While necessary in air (0.04% CO_2_), the shell is not required when cyanobacteria are in high CO_2_ levels representative of early Earth. To understand how the carboxysome shell responds to increased CO_2_ conditions, we used a Grx1-roGFP2 redox sensor and single cell timelapse fluorescence microscopy to track subcellular redox states of *Synechococcus* sp. PCC 7002. Comparing different levels of compartmentalization, we targeted the cytosol, a shell-less carboxysomal assembly intermediate called procarboxysomes, and carboxysomes. Carboxysome redox state was dynamic, and, under 3% CO_2_ conditions, procarboxysome-like structures formed which were only partially encapsulated and exposed the carboxysome contents to the cytosol. This work expands the adaptability of carboxysomes to environmental conditions and builds understanding of the selective forces that initially drove carboxysome evolution.

Photosynthetic bacteria in the phylum cyanobacteria are thought to have reshaped Earth by creating an oxygen-rich atmosphere ~2.4 Gya; in modern times, they are again poised to significantly alter our planet by serving as a useful carbon dioxide (CO_2_) sink in the face of climate change.^1,2^ Conducting 25% of annual global carbon fixation,^3^ cyanobacteria are still crucial to the Earth’s carbon cycle. They accomplish this feat by implementing an efficient CO_2_-concentrating mechanism (CCM) that uses carboxysomes, proteinaceous bacterial microcompartments which house their carbon-fixing machinery, ribulose-1,5-bisphosphate carboxylase/oxygenase (Rubisco) and carbonic anhydrase (CA).^4,5^ The CCM also employs numerous membrane-associated HCO_3_^−^ transporters: HCO_3_^−^ is concentrated in the cytosol, diffuses into carboxysomes, is rapidly converted to CO_2_ by CA, and ultimately creates a CO_2_-rich environment around carboxysomally-localized Rubisco. As cyanobacteria originally evolved in a relatively rich CO_2_ environment, they are thought to have developed two convergent lineages of carboxysomes, α/β, and a CCM in response to a simultaneous rise of O_2_ and fall of CO_2_ levels in the atmosphere.^1^ Debate remains in the field on the exact timing and evolutionary pressure of this process.^1,6^ While some caveats do exist, environmental CO_2_ modulation can be used to examine how cyanobacteria may react to increasing CO_2_ levels from climate change as well as explore selective pressures historically experienced by cyanobacteria.^1^

The carboxysome shell was historically thought to be selectively permeable to bicarbonate (HCO_3_^−^) while limiting diffusion of molecular oxygen and CO_2._^7–9^ However, recent computational analysis suggests that the diffusion of oxygen and CO_2_ is remarkably unrestrained by the shell and that diffusion limitation is largely restricted to cations and larger molecules.^9–11^ Further investigation is needed to identify which molecules, beyond carbon fixation metabolites, are subject to diffusion limitation by the carboxysome shell and to elucidate the functional significance of this selective permeability.

*De novo* formation of β-carboxysomes is initiated by aggregation of Rubisco and CA to the pole of the cell via the scaffold protein, CcmM, into a structure known as the procarboxysome.^12–19^ Procarboxysomes are transient and short-lived intermediates preceding full encapsulation of the shell and maturation into carboxysomes.^13,20^ Little is known on the permeability and functional state of procarboxysomes. However, because the shell is either absent or not yet fully formed, procarboxysomes would have greater permeability than a carboxysome.^21^ Procarboxysomes can be studied in a perpetual state utilizing shell knock-out mutants. In many cyanobacteria, including *Synechococcus* sp. PCC 7002 (hereafter PCC 7002), the essential trimeric shell protein, CcmO, is encoded at a separate genomic locus distinct from the *ccm*-operon. The *ccm*-operon encodes other necessary carboxysome proteins including the hexameric (CcmK1, CcmK2) and pentameric (CcmL) shell proteins in addition to the aggregation proteins, CcmM and CcmN.^12^ Failure of shell assembly in CcmO knock-out lines (Δ*ccmO*) results in the terminal formation of procarboxysomes (1–2 per cell) without disrupting other core elements of carboxysomes.^12^ *ΔccmO* mutants exhibit a high-CO_2_-requiring (HCR) phenotype and are unable to grow in air (0.04% CO_2_), but can be fully rescued in elevated CO_2_ (3% CO_2_), allowing for study of procarboxysomes directly in high-CO_2_ conditions.^12,20^ By studying procarboxysomes, we can better understand carboxysome permeability during and after assembly as well as the procarboxysome as a potential evolutionary intermediate.

An integral aspect across a number of cellular processes, including the function of the CCM and carboxysome, is reduction-oxidation (redox) regulation (Fig. 1a).^17–19,23–28^ Under illumination, cyanobacterial photosynthetic machineries continually generate reactive oxygen species (ROS) through both water splitting and light energy dissipation from pigments. There are three main ROS formed, singlet oxygen (^1^O_2_), hydroxyl radicals (·OH), and hydrogen peroxide (H_2_O_2_). As ROS are both useful as internal signaling molecules and damaging to the cell, their levels must be carefully regulated.^29^ Levels of the longest-lived ROS, H_2_O_2_, are regulated via glutathione (GSH/GSSG), a non-ribosomal peptide-based antioxidant.^29^ GSH is oxidized into GSSG when exposed to H_2_O_2_ and reduced by NADPH with an enzymatic catalyst. In this way, the cytosol is maintained as a reducing environment.

Previous work has indicated that the internal redox environment of carboxysomes is an oxidizing environment.^13,24,30^ Notably, this suggests there is likely limitation in permeability across the carboxysome shell for redox agents. However, carboxysomal redox state has neither been directly compared to the cytosol nor has a specific redox pool been targeted,^13,24,30^ so much remains to be explored on the redox relationship of carboxysomes to the cytosol under variable conditions. The activity and function of several CCM proteins are known to be redox-regulated, such as one of the HCO_3_^−^ membrane transporters, SbtB/A, as way to modulate carbon uptake,^23,31^ and the scaffold proteins, CcmN and CcmM, as a way to adjust Rubisco packing during carboxysome formation.^18,19,24–26,32^ Others have been indicated as redox-sensitive, such as the shell protein, CcmK4,^25,26^ and both the large and small subunits of Rubisco, but it is unknown what these redox sensitivities achieve.^25,26^ The purpose and mechanism underlying this distinct redox environment in carboxysomes remains an area of active investigation. We hypothesize that the shell may serve an important role in maintaining a distinct redox state in carboxysomes to promote CCM function.

While not functional in PCC 7002, in some cyanobacterial strains, CcmM has an active γ-CA domain, which serves as the carboxysomal CA. This γ-CA is redox-regulated.^18,33^ Since a cytosolically located CA would disrupt the HCO_3_^−^ gradient generated by the CCM, CA must be inactivated during carboxysome formation in the cytosol.^34^ The redox regulation of γ-CA in CcmM suggests a clear mechanism by which the γ-CA is able to be inactivated in the reducing cytosol and activated in the oxidized carboxysome via disulfide bond formation. In contrast, the functional CA in PCC 7002 is a β-CA, IcfA (also known as CcaA), for which the regulation is unclear.^35,36^ While pioneering work found this β-CA to be inactivated by reducing agents,^36^ cysteines involved in disulfide bond formation and redox sensing have not been identified.^35^ As the shell-less procarboxysome stage of carboxysome formation contains cytosolically exposed CA, the *ΔccmO* strain provides a unique opportunity to study redox environment in procarboxysomes where CA activity is thought to be inhibited by reduction.

This work investigates the hypothesis that the carboxysome shell functions in maintaining a distinct redox state in carboxysomes. We track the dynamic redox changes within the cytosol, carboxysomes, and procarboxysomes in PCC 7002 during growth in air (0.04%) and 3% CO_2_ conditions. To accomplish this, we implemented previously characterized redox-sensitive GFPs (roGFP2) fused with glutaredoxin (Grx1) to specifically probe changes in glutathione redox pools (Figs. 1b and c).^13,22,30,37^ Using single-cell timelapse fluorescence microscopy under precisely controlled environmental conditions,^20,38^ we measured the relative redox states at subcellular levels in PCC 7002. The work provides the first direct analysis of the redox state of the cyanobacterial cytoplasm, highlighting differences in the redox state within carboxysomes and showing that these differences are influenced by the carboxysome shell, particularly regarding the exclusion of thiol reductants, which has important implications for the functional role of the shell in regulating carboxysomal CA during biogenesis.

Additionally, in response to elevated CO_2_ conditions, carboxysome permeability increases, as measured by redox state, which appears to be from incomplete shell encapsulation. We refer to these large, permeable structures as “procarboxysome-like” to indicate their similarity in redox state and cytosolic accessibility to carboxysome formation intermediates, procarboxysomes. This work provides novel insight into the CO_2_ concentration-based evolutionary pressures leading to the encapsulation of Rubisco and its co-localization with CA. Furthermore, this study provides a window into how cyanobacteria may adapt to anthropogenetic increases in CO_2_ levels.

## Results

### Carboxysomes are More Oxidized than the Cytosol and Procarboxysomes

**Fig 2.**
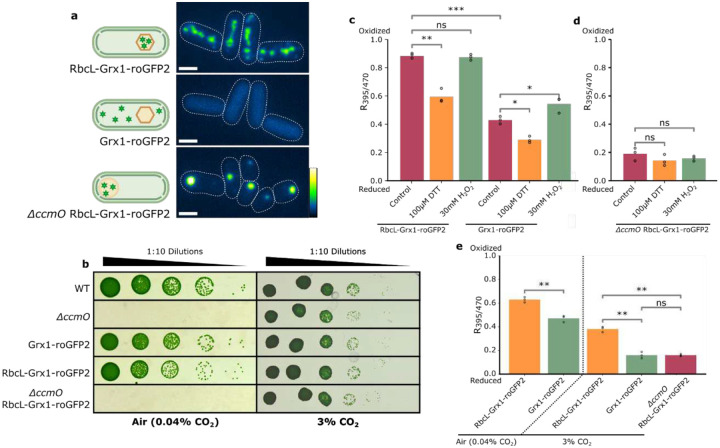
Grx1-roGFP2 Strains Characterization (A) Fluorescence microscopy images of GFP intensity and localization in exponentially growing PCC 7002 strains growing in air (RbcL-Grx1-roGFP2 and Grx1-roGFP2) or growing in 3% CO_2_ (*ΔccmO* RbcL-Grx1-roGFP2). Indicating the localization pattern of Grx1-roGFP2 to each of the subcellular regions of interest (carboxysome, cytosol, and procarboxysome, respectively). Color bar indicates GFP intensity. Scale bars represent 2μm. (B) Growth of strains in air (0.04% CO_2_) and 3% CO_2_ on 1% and 0.5% agar spot plates, respectively.^39^ RoGFP strains showed either WT or *ΔccmO*-like growth. Ten-fold serial dilutions were plated and imaged after 72 hours. Images are representative of three biological replicates. (C and D) Spectrofluorometer measurements of strains grown in bulk liquid with 100 μM DTT and 30 mM H_2_O_2_ added 25s prior to measurement. (C) Grown in air, carboxysomes (RbcL-Grx1-roGFP) and the cytosol (Grx1-roGFP) are reduced when exposed to DTT whereas only the cytosol is oxidized with exposure to H_2_O_2_. (D) Grown in 3% CO_2_, procarboxysomes (*ΔccmO* RbcL-Grx1-roGFP2) do not change redox state under either condition. (E) Spectrofluorometer measurements of strains grown in bulk liquid in air and 3% CO_2_. Carboxysomes (RbcL-Grx1-roGFP) are more oxidized than the cytosol (Grx1-roGFP) and procarboxysomes (*ΔccmO* RbcL-Grx1-roGFP2) and both the cytosol and carboxysome become more reduced under 3% CO_2_ than in air. No data is included for the procarboxysome redox state under air as *ΔccmO* RbcL-Grx1-roGFP2 cannot grow in air. Cultures were grown in respective CO_2_ conditions for 24 hours prior to measurement. The results are representative of three biological replicates, n = 3, and data was analyzed by Student’s t-test. *, p < 0.05; **, p < 0.01.

To probe subcellular cyanobacteria redox environments, we expressed Grx1-roGFP2^22^ either as soluble protein to target the cytosol or as an C-terminal translational fusion with the large subunit of Rubisco (RbcL) to target carboxysomes/procarboxysomes (Fig. 2a). Live-cell fluorescence imaging showed that the roGFP signal morphology and localization for carboxysomes, cytosol, and procarboxysomes was consistent with previous literature.^12,20,38^ To assess unintentional disruption of carboxysome function or shell structure, we tested strains for lack of a high-CO_2_ requiring phenotype.^12,20,39^ Addition of RbcL-Grx1-roGFP2 construct to the *ΔccmO* mutant did not disrupt the existing *ΔccmO* high-CO_2_ requiring growth pattern^12,20^ while all other strains had WT-like growth in both air (0.04% CO_2_) and 3% CO_2_ (Fig. 2b). Overall, the roGFP-expressing strains appeared to be functionally comparable to WT and previously characterized *ΔccmO* mutant^12^ with the growth patterns (Fig. 2b) and western blots (Fig. S1) indicating RbcL-Grx1-roGFP2 addition did not cause excessive overexpression or disrupt normal carboxysome function or localization.

To confirm sensitivity of the roGFP probe, oxidizing (H_2_O_2_) and reducing agents (DTT) were added to bulk cultures and the fluorescence emission spectra of roGFP was measured, generating ratiometric readouts of the redox environment in each strain (Fig. 2c). The cytosol showed consistent redox responses whereas carboxysomes were unresponsive to H_2_O_2_ addition, indicating either differences in shell permeability of the oxidizing and reducing agents or that carboxysomes were already fully oxidized.^30^ No significant changes were measured in procarboxysomes with either redox agent, likely due to the preexisting reduced state from growing in 3% CO_2_ limiting DTT impact and lower sensitivity to H_2_O_2_ as seen in carboxysomes (Fig. 2d). Because the roGFP probe relies on a ratiometric measurement, the measurement of redox state is independent of roGFP concentration, subsequently allowing for comparison of strains with differing GFP signal intensity.^22^ This feature of the roGFP system is supported by comparable redox readouts of the diffuse cytosol signal and the cytosolically exposed procarboxysome with high intensity puncta (Fig. 2a and e). These results indicate that the roGFP system is functional at a bulk culture level and can be used to probe the redox poise of different subcellular regions.

To observe if subcellular redox states respond to CO_2_ concentration changes, the redox state was measured in bulk cultures grown either in air (0.04% CO_2_) or 3% CO_2_ (Fig. 2e). Carboxysomes were more oxidized than the cytosol in both air and 3% CO_2_ (Fig. 2e). However, unexpectedly, both the cytosol and carboxysomes became more reduced in 3% CO_2_ conditions compared to their air-grown counterparts. Although previous work has probed the carboxysome environment independent of the cytosol,^13,30^ this is the first time, to our knowledge, that the redox environment of these two subcellular regions have been directly compared. The procarboxysome redox state was not significantly different than the cytosol in 3% CO_2_. This supports that procarboxysomes, with their non-existent shell,^12^ are exposed to the similarly reduced cytosolic environment. Furthermore, CO_2_ concentration impacts redox environment in subcellular regions of PCC 7002.

### Carboxysome Redox State Dynamically Responds to [CO_2_]

**Fig 3.**
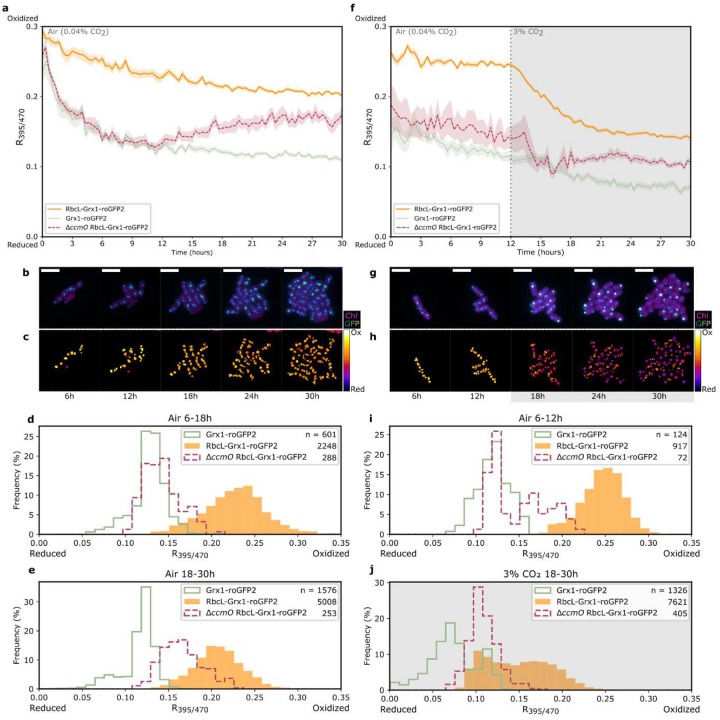
Redox state dynamics under modulated [CO_2_]. Aggregated redox state from timelapse fluorescence microscopy of carboxysomes (RbcL-Grx1-roGFP2), procarboxysomes (*ΔccmO* RbcL-Grx1-roGFP2), and cytosol (Grx1-roGFP2) over 30 hours of growth in (A-E) air or (F-J) 3% CO_2_ conditions from hour 12 to 30. (B and G) GFP and chlorophyll fluorescence and (C and H) ratiometric images of representative carboxysomes over 30 hours of growth. Redox color bar spans from *R*_395/470_ 0 to 0.3. Histograms represent frequency of redox state of each subcellular region when growing in air (D, E, I) or 3% CO_2_ (J). Wildtype background fluorescence was subtracted from excitation intensity values at 395 nm and 470 nm (emission at 520 nm). Error bars represent standard error with n changing over the course of the experiment for each strain as a result of cell division (Fig. S3). Scale bars represent 5μm. Representative data from three biological replicates.

The bulk culture results revealed that the redox state in cyanobacteria responded to changes in [CO_2_]. To investigate this response to 3% CO_2_ at a finer scale, we used time-lapse microscopy to capture the redox dynamics of subcellular regions of PCC 7002. This approach allowed for simultaneous comparison of redox dynamics across a population as well as individual cell responses. In agreement with the bulk data, carboxysomes were consistently more oxidized than the cytosol and procarboxysomes (Fig. 3a–e, Movie S1 and S2). Both the cytosol and carboxysomes become more reduced over time, whereas procarboxysomes exhibit an intermediate redox state, especially in air (0.04% CO_2_) where this mutant is unable to grow and may be experiencing photodamage. When the growing conditions were changed from air (0.04% CO_2_) to 3% CO_2_, carboxysomes shifted to more reduced redox environment over 8 hours (Fig. 3f–j, Movie S3 and S4). However, when reversed from 3% CO_2_ back to air (0.04% CO_2_), the average carboxysomes steady-state exhibited hysteresis and did not return to the same pre-high CO_2_ redox state (Fig. S2, Movie S5 and S6). These observations suggest that the redox state in carboxysomes is dynamic, but, at the average population level, did not explain what might drive these shifts.

### Procarboxysome-Like Structures Form in 3% CO_2_

**Fig 4.**
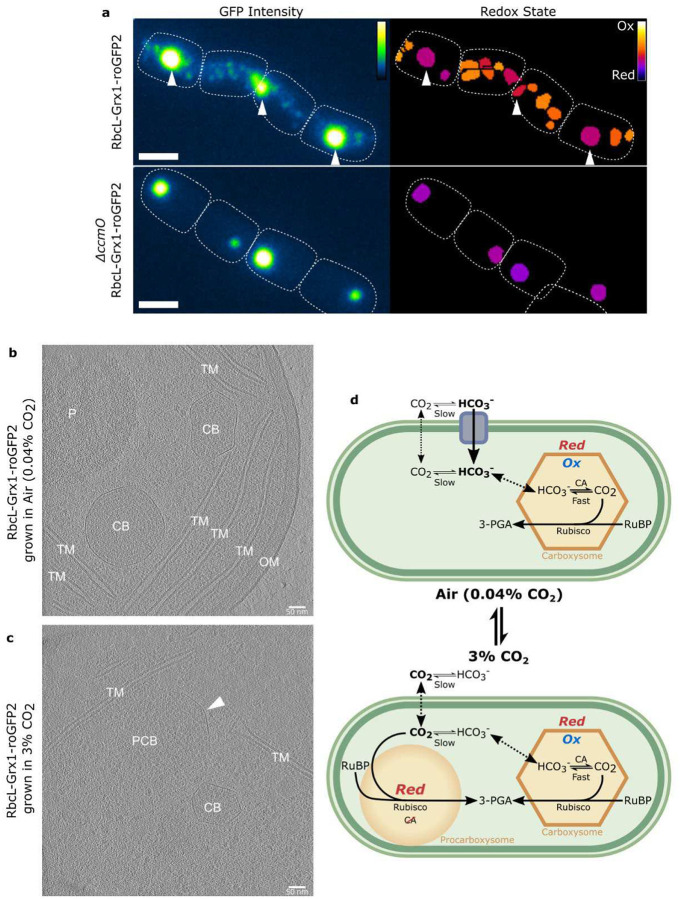
Procarboxysome-like structures form in 3% CO_2_. (A) GFP fluorescence microscopy showing that under 3% CO_2_ there are procarboxysome-like puncta present in carboxysome-labeled strain (RbcL-Grx1-roGFP2). These procarboxysome-like structures have a similar size, fluorescent intensity, and redox state as procarboxysomes as seen in the procarboxysome-labeled strain (*ΔccmO* RbcL-Grx1-roGFP2). Color bars indicate GFP intensity and redox ratio (0–0.5) respectively and scale bars represent 2μm. CryoET tomograms of carboxysome-labeled strains (RbcL-Grx1-roGFP2) grown either under (B) Air or (C) 3% CO_2_. Arrow indicates where the carboxysome shell is only partially surrounding the procarboxysome-like structure of aggregated Rubisco. (D) Proposed dynamic cyanobacterial CCM model under low and high CO_2_ conditions. Under reducing environments carbonic anhydrase (CA) activity is thought to be inhibited,^36^ and bicarbonate (HCO_3_^−^) transport downregulated as the pH shift in 3% CO_2_ reduces [HCO_3_^−^]. CB: Carboxysome, PCB: Procarboxysome/procarboxysome-like structure, TM: Thylakoid Membranes, OM: Outer Membranes, P: Polyphosphate Body.

Further analysis of the population abundances of subcellular redox states revealed that, in 3% CO_2,_ carboxysomes in RbcL-Grx1-roGFP2 have a bimodal distribution (Fig. 3j) which disappears when returned to air (Fig. S2). In addition, we also noticed changes in the morphology of the fluorescent puncta labeling carboxysomes in RbcL-Grx1-roGFP2. When grown in 3% CO_2_, there was formation of large, high intensity signal puncta with redox states comparable to the procarboxysome puncta in *ΔccmO* RbcL-Grx1-roGFP2 (Fig. 3, Fig 4a) which we refer to hereon as procarboxysome-like structures. Once the cells were returned to air, the reduced state of these procarboxysome-like structures persisted for <6 hours before presumably being processed into carboxysomes (Fig. S2). Additionally, similarly large, high intensity signal puncta were observed in CcmK1-GFP and CcmN-GFP labeled strains grown in 3% CO_2_ (Fig. S4)

Given that procarboxysomes and procarboxysome-like structures share a similar redox state with the cytosol (Fig. 3j, S2) and that procarboxysome do not have shells,^12^ we therefore concluded that the procarboxysome-like structure’s shell must also have greater permeability to cytosolic reducing agents. In support, CryoET imaging revealed that these procarboxysome-like structures possess incomplete, partially assembled shells that failed to fully encapsulate Rubisco and form closed microcompartment (Fig. 4c, Movie S8), in contrast to fully encapsulated carboxysomes (Fig. 4b and c, Fig. S5, Movies S7 and S9) or shell-less procarboxysomes in control strains (Fig. S5, Movie S10).

## Discussion

The function and permeability of the carboxysome shell has been an area of debate for over thirty years.^34,36^ One leading hypothesis was that the shell served as a barrier to CO_2_ and oxygen,^7,8,15^ thus trapping CA-derived CO_2_ with Rubisco and limiting Rubisco’s side reaction with oxygen. However, recent computational work suggests that CO_2_ and oxygen diffusion is only minimally limited by the carboxysome shell.^9–11^ Another possibility is that the shell serves to maintain a pH differential,^10,40,41^ though modeling of diffusion argues that protons would be able to freely cross the shell and dissipate pH differences.^7,9,42^ Consistent across all these studies is that larger molecules, such as the reactant RuBP, experience selective permeability across the shell pores with potential limitations to size passage and favorability to anions.^7,9,13,43,44^ Additionally, one key chemical environmental difference of the carboxysome lumen has been identified prior and here, which is that carboxysome are more oxidized than the cytosol.^13,30^ When the shell is intact *in vitro*, reduction by reducing agents, such as TCEP^21^ and DTT^36^, occurs on the order of tens of minutes compared to carboxysomes with disrupted shells.^21^ From this, we support the theory that the carboxysome shell serves as a barrier to redox compounds, such as glutathione and NADPH, to create a distinct internal oxidizing environment.^18^

The redox state of carboxysomes may serve as a chemical “switch” to indicate completion of the shell and activate its carbon-fixing role in the CCM.^13,32^ Oxidation of CcmM promotes homodemixing, which is thought to create metabolite channels within carboxysomes and promote access for Rubisco repair proteins.^24^ Disruption of the disulfide bonds in CcmM and CcmN resulted in fewer, large, aberrant carboxysome structures with a HCR phenotype.^19,32^ CA activity across several cyanobacteria species have also been shown to be activated by oxidizing conditions,^18,33,36^ and, critical to CCM function, remain inactive when in a reducing environment such as the cytosol.^34^ The exact mechanism of redox (in)activation of β-CA needs to be more deeply explored in PCC 7002.^36^ This theory begs the question: if the need of the carboxysome shell is removed by growing in high CO_2_, are carboxysomes maintained as an oxidized environment?

This work represents the first exploration on the impact of CO_2_ concentration on the carboxysome redox environment. We found that carboxysomal redox state is dynamic and becomes more reduced in high CO_2_. This appears to be a result of changes in the shell structure increasing carboxysome permeability to redox agents as the changing inorganic carbon pool simultaneously affects global redox metabolism.

This dynamic redox state brings up the unanswered question of what drives and maintains carboxysome oxidation state in the first place. While redox regulation has repeatedly been implicated in controlling γ-CA activity^18,33^ and carboxysome aggregation via CcmM structure and binding affinity,^18,19,24^ to our knowledge, there has not yet been an identified component of the carboxysome system capable of actively oxidizing the internal carboxysome environment during carboxysome formation. In other bacterial microcompartments (BMCs), the encapsulated reactions rely on the NAD(P)H electrochemical cycle. In these BMCs either reductases are co-encapsulated or FeS clusters are thought to occupy shell pores to transfer elections across the BMC shells.^45,46^ These mechanisms also support that the BMC shell is a barrier to redox agents such as NADH. For carboxysomes, we speculate that the diffusion of oxygen and potentially H_2_O_2_ across the shell gradually oxidizes the trapped glutathione pool in the carboxysome lumen, as seen in purified α-carboxysomes.^30^ This pool cannot be reduced due to shell impermeability to reducing agents and protein reductases.

None of the known carboxysome components have been reported to possess enzymatic oxidizing capability. It is possible that the presence of the roGFP sensor alters the redox environment in which it is located and artificially creates an oxidized carboxysomal environment but, given the evidence for redox regulation of Cas, CcmM, and CcmN,^18,19,24,32^ there is biologically based functional support for carboxysomes being an oxidized environment. As this study uses a glutaredoxin to specifically track the glutathione pool, further work targeting other forms of redox activity in carboxysomes is warranted, such as other redox pools (NADPH) and reductase-dependent methionine oxidation.^47^ Other redox probes could be implemented but, given the unknown permeability of the carboxysome shell to chemical probes, such as SNAP dyes,^13^ this nanometer-scale subcellular region^48^ remains challenging to study.

There was a consistent shift in redox environment the first 2–3 hours, likely a consequence of the cells adjusting to the environmental conditions of the microscope and therefore was disregarded in the CO_2_ modulation data.^39^ However, this shift still has intriguing implications. Because the state of carboxysomes appears to start as oxidized and then trend towards a more reduced steady state over time (Figs. 3a and f, S2), there may be some adjustment of the redox state of the carboxysomal glutathione pool. There is potential for more subtle permeability modification of carboxysomes to redox agents, perhaps through the less explored shell proteins with larger pores such as CcmP.^7^ The shell is not completely impermeable to reducing agents, such as DTT (Fig. 2c) or TCEP^21,30^. Further, it is unclear if the shell acts as a diffusion barrier to H_2_O_2_ (Fig. 2c). We speculate that the lack of response to H_2_O_2_ may be that the cytosol serves as a buffer against this oxidizing agent or that carboxysomes are already fully oxidized, as found in previous in vitro studies.^21,30^ We also speculate that the formation of aggregated cells may affect gas exchange or other physiologically significant parameters leading to a more reduced condition, which may drive the trend towards reduction, but this will need a more thorough analysis as part of a future study. Further work is needed to explore carboxysome redox dynamics, shell permeability to redox agents, and processes driving carboxysome oxidation.

Discovery of partially shelled, reduced procarboxysome-like structures in high CO_2_ conditions provides both powerful insights into carboxysome evolution and function and opens new questions. Unique to the work presented here, we leveraged the terminal procarboxysomes in *ΔccmO* mutants to directly compare similarities in morphology and redox state of procarboxysome-like structures forming high [CO_2_] in non-knockout cells. Procarboxysomes and procarboxysome-like structures share a reduced state, like the cytosol, and have missing or incomplete shells respectively (Fig. 4a). Recent work studying *in vitro* α-carboxysomes found that carboxysomes were larger and shell proteins had increased fluidity in reducing conditions.^49^ Additional experiments, such as tracking shell development over time,^12,50^ and FRAP of shell proteins to determine mobility^49^ would be needed to understand the development of the carboxysome shell in these procarboxysome-like structures. Given there is not an impact on growth by the roGFP strains (Fig. 2b), western blots show minimal excess RbcL in mutant strains (Fig. S1), and similar puncta are observed in other carboxysome protein-labeled strains (Fig. S4), it is unlikely that procarboxysome-like structures are the result of aberrant Rubisco aggregations.^51^ Rubisco has been previously observed to have differential localization in response to environmental conditions.^52^ Increased permeability from incomplete carboxysome shells would result in moderate increase of photorespiration rates,^28,53^ but given that previous studies with carboxysome mutants were conducted in high CO_2_, it is difficult to determine if these WT photorespiration rates were elevated as well if procarboxysome-like structures were present under these conditions. Future work is needed to explore the exciting details of these dynamics.

Notably, after six hours of being returned to air, reduced procarboxysome-like structures appear to either progress into oxidizing carboxysomes or are diluted by the formation of *de novo* carboxysomes (Fig. S2). The data presented here is insufficient to determine between whether this process is driven by changes in carboxysome protein expression levels or structural alternations in response to redox shifts.^19,24,49^ There is a lack of agreement in the literature on if the expression of carboxysomal proteins in high CO_2_ conditions is increased,^48^ decreased,^54^ or remains the same.^55^ While additional RbcL from the expression of the RbcL-Grx1-roGFP2 construct under its native promoter^20^ may also alter carboxysome formation, the fusion protein does not appear to increase total RbcL levels relative to WT (Fig. S1) and we do not note any growth rate reduction that would be indicative of excess protein expression and aggregation (Fig. 2b).^51^ Mutation of the disulfide bonds in CcmM in *Synechococcus* sp. PCC 7942 resulted in formation of large, HCR carboxysomes.^19^ This suggests the mechanism that reduction of CcmM may alter carboxysome condensation and shell formation leading to the larger, more permeable procarboxysome-like structures observed in high CO_2_, and is a reversable process when CcmM becomes oxidized again.

High CO_2_-specific formation of procarboxysome-like structures could point to procarboxysomes as an evolutionary intermediate during changing CO_2_ conditions.^1,40^ Pioneering studies on *Synechococcus* sp. PCC 7942^56^ and *Synechocystis* sp. PCC 6803^48,55^ found there were fewer carboxysomes per cell when grown in increased [CO_2_]. When *Synechococcus* UTEX 625 was grown in 5% CO_2_, a subset of carboxysomes were larger and irregularly shaped,^57^ indicating that formation of procarboxysome-like structures in high [CO_2_] is not a strain specific phenomena.

We hypothesize that the carboxysome shell serves as a diffusion barrier to redox agents, such as glutathione, in order to maintain an oxidizing environment in air (Fig. 4b).^18^ In high CO_2_, an incomplete shelled procarboxysome-like structure with greater permeability to the cytosol would allow Rubisco to remain exposed to the high cytosolic CO_2_ without needing the CCM and minimal to absent CA activity.^58,59^ This work paves the way for a more detailed understanding of carboxysome formation, shell permeability, and redox regulation of carbon fixation. By better understanding these processes, we can more effectively implement carboxysomes for applications in biotechnology as well as guide research on the pressures driving carboxysome evolution.

## Methods

### a. Strain cultivation

PCC 7002 strains were cultivated in AL-41 L4 Environmental Chambers (Percival Scientific, Perry, IA) at 37°C under constant illumination (~150 μmol photons m^−2^ s^−1^) by cool white, fluorescent lamps, under either ambient (air, 0.04%) or elevated (3%) CO_2_ conditions. Cultures were grown in 25 ml of A+ media in orbital shaking baffled flasks (125 ml) contained with foam stoppers (Jaece Identi- Plug), or on pH 8.2 A+ media solidified with Bacto Agar (1%; w/v). Antibiotics were added for routine growth of strains (kanamycin, 100 μg/ml; gentamycin, 30 μg/ml).

### b. Plasmid and strain construction

All plasmids and strains used in this work are described in Table S1 and Table S2. Plasmids were created through Gibson assembly of plasmid backbones (pUC19) and PCR-amplified inserts, generated using Phusion polymerase (Thermo Fisher Scientific) and primers described in Table S3. Cyanobacterial strains were generated by transforming cells in exponential/early linear growth phase with 0.5 ng/ml of plasmid DNA, containing the insert of interest flanked by 600–base pair homology arms for recombination into a specified genomic locus. After incubation at 30°C in constant illumination (50 to 150 μmol photons m^−2^ s^−1^) for 24 hours, transformed cells were selected for with appropriate antibiotic on plates in ambient CO_2_, for non- high-CO_2_ requiring strains, and 3% CO_2_ for high-CO_2_ requiring strains, respectively. From plates, individual colonies were patched onto new plates and tested for segregation. Confirmation of segregation was confirmed by PCR, using primers specific for *glpK*. Presence of the insert-specific PCR product and absence of the WT-specific PCR product was used as an indicator of full segregation.

### c. Spot plating

The growth of PCC 7002 was measured on agar plates as described. Plates at 0.5 and 1% agar were spotted with strains in triplicate. Liquid cultures of each PCC 7002 strain were diluted to 0.05 OD_730_ and five 1:10 serial dilutions were performed. Five μL of the serial dilutions wereused for each spot and allowed to dry (30 min) prior to incubation. Images were taken 3 days after spotting the plates with a backlight on a Kaiser eVision light plate and imaged with a Nikon D7200 digital single-lens reflex camera.

### d. Liquid Growth Curves

The growth of PCC 7002 was measured in liquid cultures as described. The precultures were started from PCC 7002 cells scraped from plates and grown in the same conditions as the growth curve cultures, either ambient (air, 0.04%) or elevated (3%) CO_2_ conditions. 50mL A+ cultures were inoculated in triplicate with 1 mL of PCC 7002 pre-culture diluted to 0.14 OD_730_ and grown in the standard conditions described in Strain Cultivation. During the growth curve, time points were taken every 24 hours for 72 hours. At each time point, 200 μL was removed from each culture and the OD_730_ was measured in a 96-well plate on a Tecan Spark multimode microplate reader.

### e. Spectrofluorometer

#### i. Chlorophyll quantification

50mL cultures were inoculated from pre-cultures grown in liquid cultures. Liquid cultures were grown to OD_730_ 0.3–1.0 in either ambient (air, 0.04%) or elevated (3%) CO_2_ conditions. Chlorophyll was methanol extracted from 1 mL of culture diluted to 0.3 OD_730_ as described in Porra et al..^60^ Absorbance at 665 nm was measured and the chlorophyll content was calculated with equation 1.


Equation 1: $$Chl\;a\left(\frac{\mu g}{mL@0.233\;{OD}_{730}}\right)=16.29*Abs@665\;nm$$

#### ii. Fluorescent Spectra Measurement

Once each culture’s chlorophyll had been quantified, each original culture was diluted to a chlorophyll concentration of 3 μg/mL in A+ media. The normalized chlorophyll cultures were loaded into a FireflySci 1FLPS Disposable Cuvette. Fluorescence was measured using a Fluorolog-3 spectrofluorometer (Horiba Jobin Yvon). Grx1-roGFP2 was excited from 350- to 480 nm with a 5 nm slit and a step size of 1 nm and the fluorescence emission spectra was gathered with an emission wavelength of 510 nm with a 5 nm slit. For sensitivity tests, 30 mM H_2_O_2_ or 100 μM DTT was added to the cuvettes and allowed to incubate for 30 s prior to measurement.

#### iii. Ratiometric Data Processing

In replicates of three or four, WT emission was averaged at excitation at 395- and 470 nm respectively (b_395_ and b_470_). This value was then subtracted from each Grx1-roGFP2 strain’s emission value from 395- and 470 nm excitation respectively (I_395_ and I_470_) before dividing the emission from 395 nm excitation by the emission from 470 nm excitation (equation 2) and averaging across samples.


Equation 2: $$R_{395/470}=\frac{I_{395}-b_{395}}{I_{470}-b_{470}}$$

### f. Quantitative microscopy

Fluorescence images were taken using a customized Nikon TiE inverted wide-field microscope with a near-infrared–based Perfect Focus System.^20,38^ Temperature and CO_2_ concentrations were controlled with a Lexan environmental chamber outfitted with a ProCO_2_ P120 Carbon Dioxide Single Chamber Controller (BioSpherix, Parish, NY), and growth light was controlled via a transilluminating red light emitting diode (LED) light source (Lida Light Engine, Lumencor, Beaverton, OR). A highspeed light source with custom filter sets was used for imaging Spectra X Light Engine, Lumencor, Beaverton, OR), along with a hardware-triggered and synchronized shutter for control of imaging and growth light. NIS Elements AR software (version 5.11.00 64-bits) with Jobs acquisition upgrade was used to control the microscope. Image acquisition was performed using an ORCA-Flash4.0 V2+ Digital sCMOS camera (Hamamatsu) with a Nikon CF160 Plan Apochromat Lambda 100× oil immersion objective (1.45 numerical aperture).

For long-term time-lapse microscopy, cells in exponential or early linear phase were diluted to 0.14 OD_730_, all strains were mixed in equal proportions, and 1 μL was spotted onto a 1% agarose A+ pad. Cells were dried onto the pad (20 min), inverted onto a 35-mm glass bottom imaging dish (ibidi), which was then wrapped in parafilm to keep the pad from drying out, and preincubated at 37°C for 1 hour in the dark. No antibiotics were included on the agarose pad. Images were taken every 20 min using a 395-, 470-, 555- and 640 nm LED light source Spectra X) and emission wavelengths were collected using standard GFP (395- and 470 nm excitation, 520 nm emission), RFP (595 nm emission), and Cy5 (705 nm) filters (Nikon). Cells were constantly illuminated with red light except during fluorescent imaging.

### g. Image processing and analysis

Cell segmentation was performed using MATLAB version R2020b as outlined previously.^38^ To segment (identify) individual cells, we also captured images in bright field, with the red growth light as an illumination source. Cells were then identified by applying an intensity threshold and watershed algorithm to create a cell mask. Manual mask correction was then performed to correct mistakes before data analysis. Cells that died or overlapped were removed from the mask and subsequent data analysis. Carboxysome and procarboxysome puncta were further segmented based on their GFP signal. Note that these mask images were only used for cell segmentation—reported data were measured from the original images.

Each cell’s strain was visually identified. Puncta smaller than 62 pixels in the *ΔccmO* mutant were excluded from analysis to limit misidentified puncta from background noise. Averaged intensity of WT was used for background subtraction for 395- and 470 nm excitation channels from the averaged intensity of each cell or puncta. To account for low signal in the 470 nm excitation channel, any cell or puncta that was below zero after background subtraction was brought to zero for subsequent calculations. Redox states were calculated across all strains using Equation 2. This ratio was overlayed on the respective cell or puncta mask to generate ratiometric images for ease of visualization.

For more images of the data used in this work, the authors refer the reader to the machine learning cell segmentation tool described in Huffine et.al. 2025.^61^

### h. Statistics

For the statistical comparison of *R*_395/470_ for bulk culture redox state, unpaired two-tailed Student’s t-tests were used. P values are indicated by asterisks; *p < 0.05, **p < 0.001, ***p < 0.0001.

### i. CryoET

#### i. Sample vitrification with High-Pressure Freezing

Overall high pressure freezing and waffle CryoET sample preparation was performed per standard protocol.^62^ Briefly, Quantifoil R2/2 mesh 200 holey carbon grids were glow-discharged for 45 seconds at 15 mA and placed between polished Type B planchettes. Prior to freezing, cyanobacterial cells were concentrated at 10000xg to form a cellular paste and applied to the cryo-grid using clean spatula. Sample was frozen using Wohlwend Compact 01 HPF at 2,100 bar and rapidly transferred to liquid nitrogen. Clipped cryo grids were transferred to an Aquilos dual-beam FIB-SEM microscope (Thermo Fisher Scientific) equipped with a cryo-transfer system and a 360° rotatable cryo-stage.

#### ii. Waffle sample thinning

Before milling, samples were sputter-coated with platinum (1 kV, 20 mA, 25 seconds at 0.10 mbar) to improve conductivity and reduce charging artifacts. Additional organometallic platinum was deposited using the gas injection system (GIS) operated at 28°C with a 7 mm stage working distance and 90-second gas injection time to provide protection during the milling process. Lamella preparation was performed using a series of milling steps according to the protocol^62^ using provided milling templates and sequentially decreasing ion beam currents. Initial rough and medium milling was conducted at 1 nA followed by final and finer milling at 0.5 and 0.3 nA respectively. Final polishing was performed at 30 pA and 10 pA to achieve a final lamella thickness of approximately 150–200 nm. Finally, a notch pattern was milled with the defined dimensions close to the edge of the lamella. The fabricated lamellae were positioned perpendicular to the grid plane at angle of 20° relative to the grid surface to maximize the observable area within the bacterial cells.

#### iii. Cryo-ET data collection

Lamellae were imaged using a 300 kV Titan Krios G3i transmission electron microscope equipped with a Selectris energy filter and Falcon 4i electron detector (Thermo Fisher Scientific). Tilt series were collected using SerialEM software with a dose-symmetric tilt scheme ranging ±55° with 3° increments around the pre-tilt angle defined by the milling angle, resulting in a total of 57–60 projections per tilt series. Images were acquired at a nominal magnification of 86,000×, corresponding to a pixel size of 1.965 Å at the specimen level. The cumulative electron dose for the tilt series was kept at approximately 60–80 e^−^/Å^2^. Defocus values ranged from −2 to −6 μm, and energy filtering was performed with a 10eV slit width.

#### iv. Tomogram reconstruction

Collected tilt series were reconstructed and were motion corrected using MotionCor2 software and stacked with IMOD. Tomograms were reconstructed at bin6 using AreTomo2 package with following parameters: -VolZ 1700.0 -AlignZ 1100.0 -OutBin 6 -DarkTol 0.1 -FlipVol 1 -Kv 300 -PixSize 1.965 -Wbp 1 -Patch 4 4 -TiltAxis 84.21 -TiltCor 1.

## Supplementary Material

1

This is a list of supplementary files associated with this preprint. Click to download.

- SupplementaryMovie1Lcom.avi
- SupplementaryMovie2LRatiocom.avi
- SupplementaryMovie3LHcom.avi
- SupplementaryMovie4LHRatiocom.avi
- SupplementaryMovie5HLcom.avi
- SupplementaryMovie6HLRatiocom.avi
- SupplementalMovie7roGFPairTomo.avi
- SupplementalMovie8dCcmO3CO2Tilt.avi
- SupplementalMovie9WTAirTilt.avi
- SupplementalMovie10roGFP3CO2Tomo.avi

## Figures and Tables

**Fig 1.**
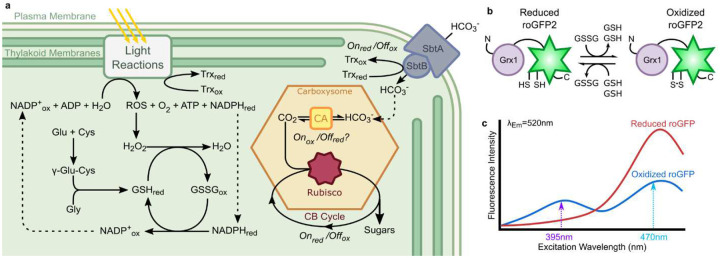
Cyanobacterial CO_2_-Concentration Mechanism (CCM) and glutathione redox roGFP2 sensor system. (A) Overview of known and theorized redox regulation in cyanobacteria. (B) Grx1-roGFP2 glutaredoxin specifically interacts with glutathione redox pools. (C) Grx1-roGFP2 fluorescence excitation spectrum with capability of ratiometric readout of redox environment.^22^ The bimodal fully oxidized spectrum has a high fluorescence ratio R_395/470_ compared to the monomodal fully reduced spectrum with a low fluorescence ratio. Emission for both excitations is collected at 520nm.

## Data Availability

Timelapse imaging data supporting the findings of this study are available within the paper and its Supplementary Movie 1–6. All data is available upon reasonable request.

## Code Availability

All code related to data analysis is available upon reasonable request.
