# Supplementary material for: [CO2] Alters Cyanobacterial Carboxysome Encapsulation and Redox State
Source: Res Sq. 2026 Feb 19:rs.3.rs-4814625. Preprint. [Version 1] doi: 10.21203/rs.3.rs-4814625/v1 (PMC12935002; doi:10.21203/rs.3.rs-4814625/v1)
Supplement: 1 [file NIHPPRS4814625V1-supplement-1.pdf]

# Supplementary Data

## Supplementary Methods

1. **Supplemental Figure 3:**
    a. **Confocal Fluorescent Imaging**
        i. For single confocal imaging, cells were grown at 37°C under constant illumination (~150 µmol photons $m^{-2}$ $s^{-1}$) by cool white, fluorescent lamps, under either ambient or high (3%) $CO_2$ to an $OD_{730}$ between 0.07-0.15. Then 1 uL was spotted on 1% agarose A+ pad with no antibiotic. Cells were allowed to dry onto the pad for 90 minutes under the same growth conditions. Cells were then inverted onto a 170-um glass bottom four chamber slide (ibidi) and imaged within 20 minutes of removing them from growth conditions. Images were taken using an Olympus Fluorview FV3000 Confocal Laser Scanning Microscope using an Olympus UPLXAPO 60X Oil Immersion Objective (NA=1.42) using standard GFP excitation at 488 nm and emission at 520 nm.
2. **Supplemental Figure 4: Western Blots**
    a. Following protocol outlined in 2021 Dahlgren et. al.[63]
    b. **Sample Preparation**
        i. Briefly, 50 mL cultures of PCC 7002 strains were grown in A+ media Stevens et al. 1973) in air or 3% $CO_2$ at 37 °C with a light intensity of 185 µmol photons m−2 s−1 for 2 days to an OD730 of 0.3-0.5. The culture was pelleted at 4300×g for 10 min at 4 °C. The supernatant was removed and cells were resuspended in 1 mL A + medium. Samples were then pelleted in a 1.5 mL tube frozen at − 80 °C for storage and to facilitate cell lysis.
    c. **Cell lysis**
        i. The cell pellet was resuspended in RIPA lysis buffer (50 mM Tris pH 7.5, 150 mM NaCl, 0.1% (w/v) SDS, 0.5% (w/v) sodium deoxycholate, 1% (v/v) Triton X-100). Cells were lysed using bead beating, with 30 cycles of 20s on and 20 s off on ice. The lysate and beads were pelleted at 2000×g and the supernatant was collected. The supernatant was then pelleted for 5 min at 15000×g and the supernatant was collected and flash frozen. The protein concentration of cell lysate was quantified using the Pierce 660 nm Protein Assay (Thermo Fisher).
    d. **Immunoblotting**
        i. 100 µg of protein was separated on a 10% SDS-PAGE gel and immunoblots were performed following the protocol from Green and Sambrook (2012). Gels prepared in parallel were imaged with Coomassie Brilliant Blue. Protein was transferred to a polyvinylidene fluoride (PVDF). After blocking membranes overnight, membranes were incubated with primary RbcL (Agrisera, cat. no. AS03037) antibodies. Membranes were then incubated with a secondary antibody conjugated to AlexaFluor 488 (Thermo Fisher, cat. no. A-11008 or cat. no. 31460). Membranes were visualized using CY2 (492nm excitation) fluorescence on an Amersham Typhoon 5 Imager.

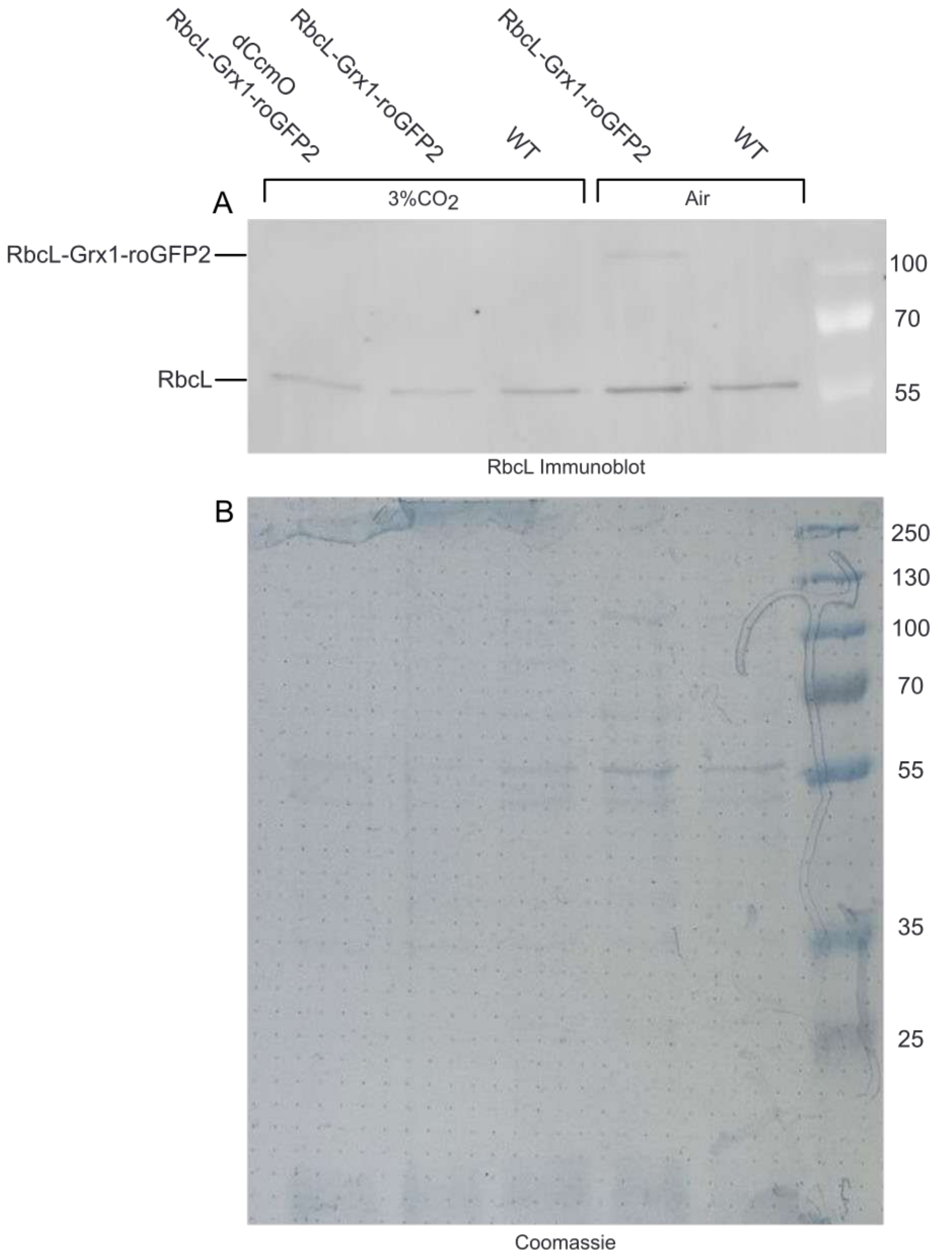


**Supplementary Figure 1. Immunoblot analysis of RbcL expression and total protein loading.** **(A)** Western blot detection of RbcL using an anti-RbcL antibody in whole-cell lysates from cells grown at 3% $CO_2$: *ΔccmO* RbcL-Grx1-roGFP2 (lane 1), RbcL-Grx1-roGFP2 (lane 2), and wild type (lane 3); and from cells grown in ambient air: RbcL-Grx1-roGFP2 (lane 4) and wild type (lane 5). **(B)** Coomassie SimplyBlue™ SafeStain SDS-PAGE gel prepared in parallel with equivalent sample volumes to demonstrate comparable total protein loading across samples.

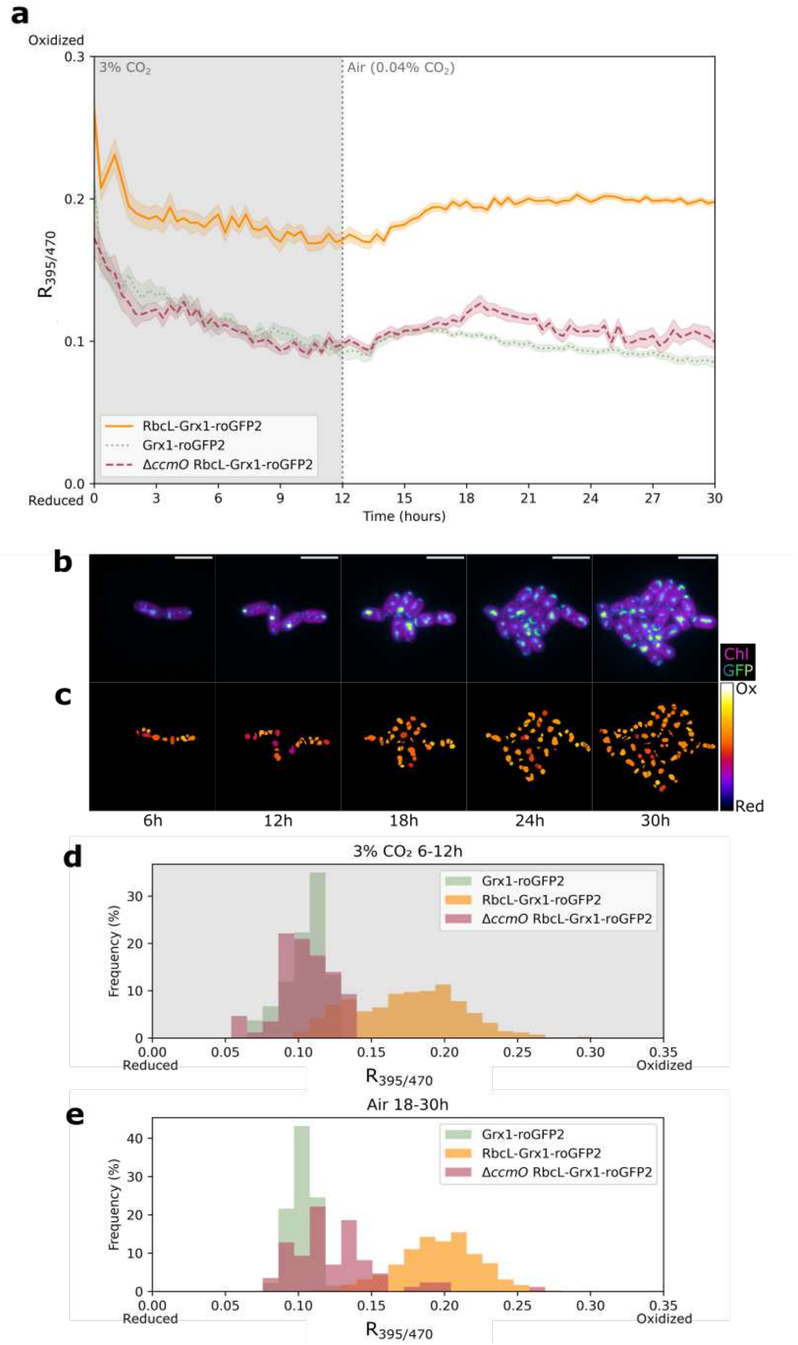


**Supplementary Figure 2. High to Low [$CO_2$] Redox Timelapse** (A) Aggregated redox state from timelapse fluorescence microscopy of carboxysomes (RbcL-Grx1-roGFP2), procarboxysomes (*ΔccmO* RbcL-Grx1-roGFP2), and cytosol (Grx1-roGFP2) over 30 hours of growth in 3% $CO_2$ conditions from hour 0 to 12. (B) GFP and Chlorophyll fluorescence and (C) ratiometric images of representative carboxysomes over 30 hours of growth. Redox color bar spans from 0 to 0.3 and scale bars represent 5μm. Histograms represent frequency of redox state of each subcellular region when growing in (D) 3% $CO_2$ or (E) air. Wildtype background fluorescence was subtracted from excitation intensity values at 395 nm and 470 nm (emission at 520 nm). Error bars represent standard error with n changing over the course of the experiment (**Fig. S3**). Representative data from three biological replicates.

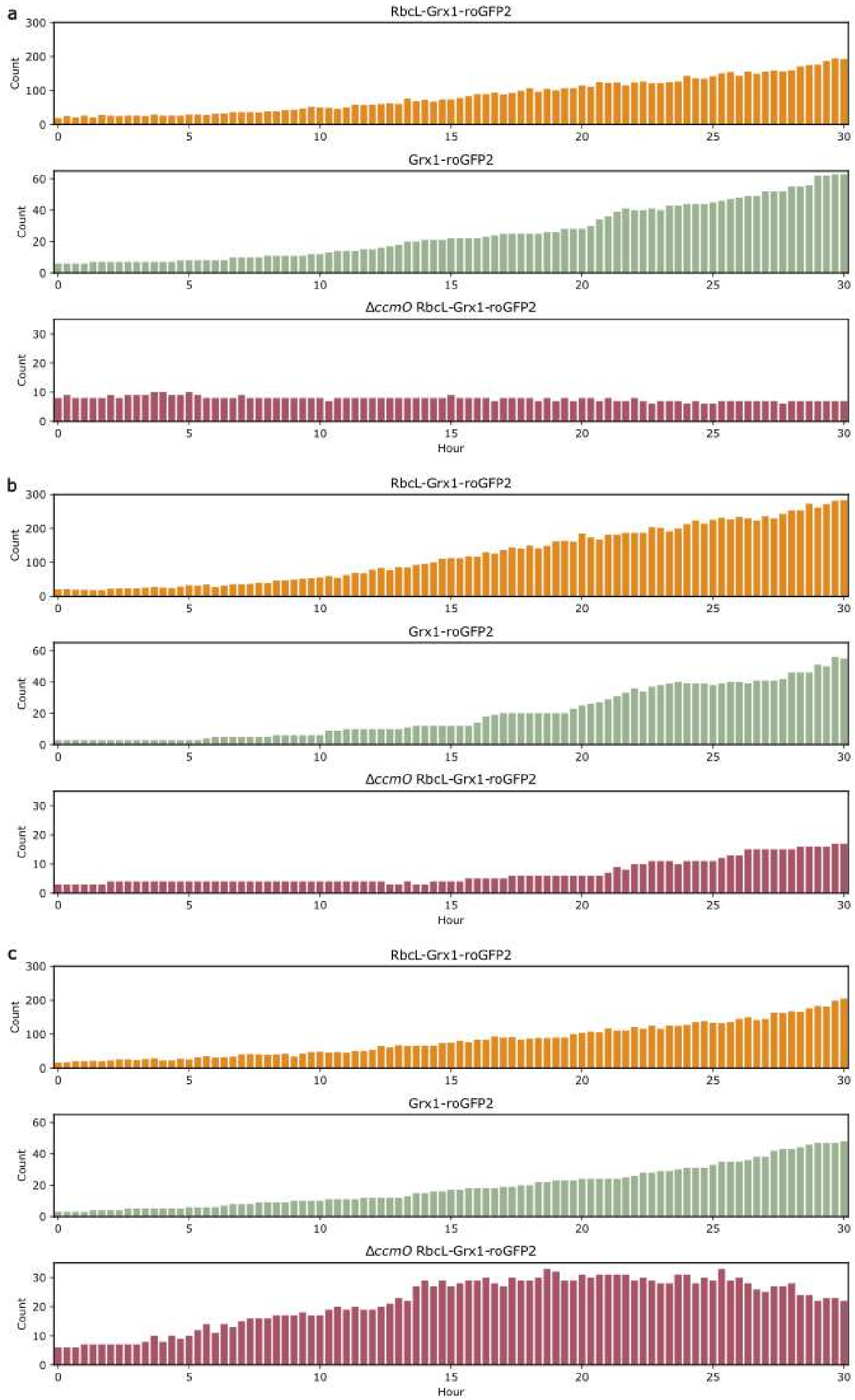


**Supplementary Figure 3. Counts for Timelapse Data** Timelapse fluorescence microscopy N-values of carboxysomes (RbcL-Grx1-roGFP2), procarboxysomes (*ΔccmO* RbcL-Grx1-roGFP2), and cytosol (Grx1-roGFP2) over 30 hours of growth (A) from Figure 3a, (B) Figure 3j, and (C) Supplementary Figure 2. Minimum N is 3.

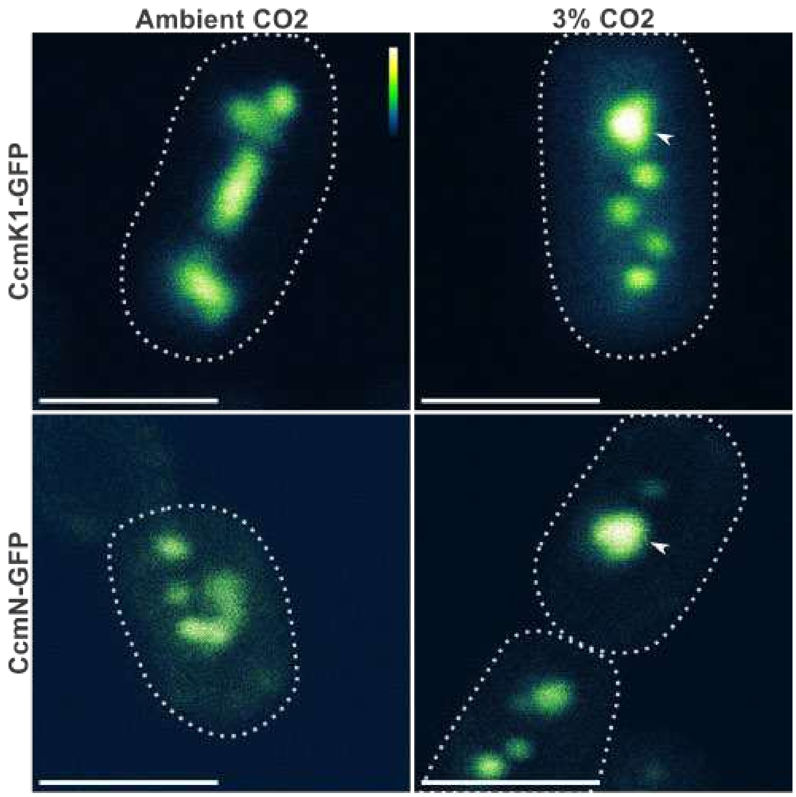


**Supplementary Figure 4. Procarboxysome-like structures in alternatively labelled carboxysomes.** Fluorescent images of CcmK1-GFP and CcmN-GFP strains grown at 3% and ambient $CO_2$. Arrows indicate larger and brighter ‘procarboxysome-like’ structures. Scale bars scale bars represent 2µm. Representative data from three biological replicates.

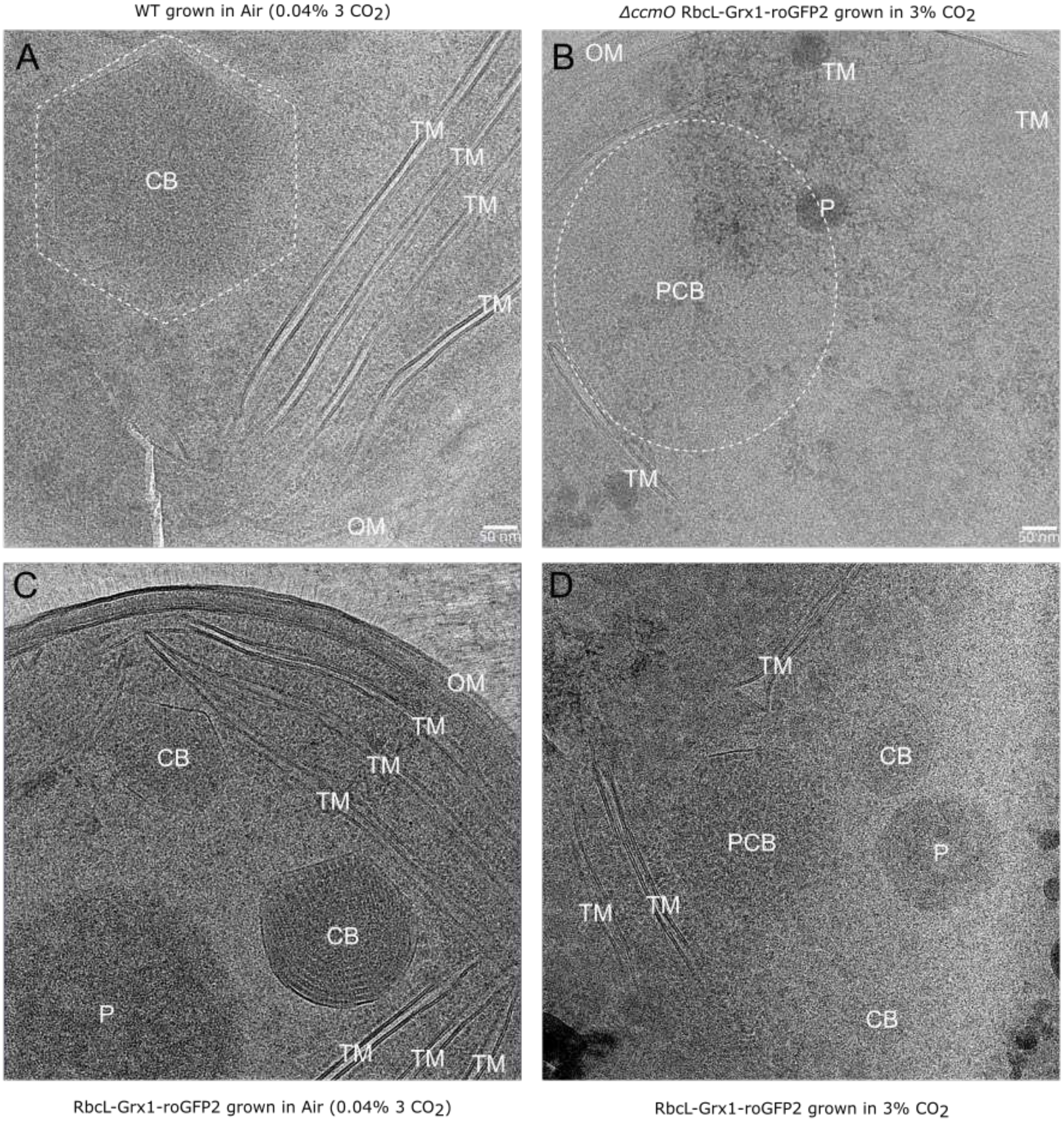


**Supplementary Figure 5. CryoET Tilt Slices.** Representative tilt images of (A)WT, (B) *ΔccmO* RbcL-Grx1-roGFP2, and (C and D) RbcL-Grx1-roGFP2 grown in either air (A and C) or in 3% $CO_2$ conditions (B and D). Rubisco is outlined in a carboxysome (A) and procarboxysome (B) for clarity. Scale bars scale bars represent 50nm. Representative data from three biological replicates. CB: Carboxysome, PCB: Procarboxysome/procarboxysome-like structure, TM: Thylakoid Membranes, OM: Outer Membranes, P: Polyphosphate Body.

**Supplementary Table 1. Strains used in this study**

| Name | Number | Description | Resistance | Reference |
|---|---|---|---|---|
| WT | - | Wild-type *Synechococcus* sp. PCC 7002 | None | - |
| Grx1-roGFP2 | scJC0501 | WT cells transformed with sJC0694 | Gm$^{R}$ | This work |
| RbcL-Grx1-roGFP2 | scJC0479 | WT cells transformed with sJC0683 | Km$^{R}$ | This work |
| *ΔccmO* | scJC0079 | WT cells transformed with sJC0132 | Km$^{R}$ | This work |
| *ΔccmO* RbcL-Grx1-roGFP2 | scJC0503 | *ΔccmO* cells transformed with sJC0691 | Km$^{R}$, Gm$^{R}$ | This work |
| *pUC19_ΔNS1_pk2-CcmK1-GFP_Gm* | scJC0520 | WT cells transformed with pUC19_ΔNS1_pk2-CcmK1-GFP_Gm$^{R}$ | Gm$^{R}$ | This work |
| *pUC19_ΔNS1_pk2-CcmN-GFP_Gm* | scJC0521 | WT cells transformed with pUC19_ΔNS1_pk2-CcmN-GFP_Gm$^{R}$ | Gm$^{R}$ | This work |

**Supplementary Table 2. Plasmids used in this study**

| Name | Number | Description | Genomic Site | Reference |
|---|---|---|---|---|
| Grx1-roGFP2-His | sJC0658 | Used as Grx1-roGFP2 PCR template to make sJC0694 and sJC0683 | - | Gutscher 2008[22] |
| Grx1-roGFP2-His_Gm$^{R}$ | sJC0694 | PK2_Grx1-roGFP2 downstream of Km$^{R}$ cassette | *glpK* | This work |
| RbcL-Grx1-roGFP2-His_Km$^{R}$ | sJC0683 | PK2_RbcL-Grx1-roGFP2 downstream of Km$^{R}$ cassette | *glpK* | This work |
| RbcL-Grx1-roGFP2-His_Gm$^{R}$ | sJC0691 | PK2_RbcL-Grx1-roGFP2 downstream of Gm$^{R}$ cassette | *glpK* | This work |
| Δ*ccmO*_Km$^{R}$ | sJC0132 | Km$^{R}$ cassette flanked by *ccmO* operon upstream and downstream homology arms | *ccmO* | This work |
| RbcL-GFP-V5_Km$^{R}$ | sJC0624 | Used as a PCR template to make cJC0683 | *glpK* | This work |
| RbcL-GFP-APEX2_Gm$^{R}$ | sJC0490 | Used as a PCR template to make cJC0691 and cJC0694 | *glpK* | This work |
| pUC19_ΔNS1:PK2_ccmK1-GFP_KmR | sJC0203 | Used as a PCR template to make sJC0701 | *NS1* | This work |
| pUC19_ΔNS1:PK2_ccmN-GFP_KmR | sJC0201 | Used as a PCR template to make sJC0705 | *NS1* | This work |
| pUC19_ΔNS1_pK2-ccaA-GFP_ Gm$^{R}$ _JABp0026_v1 | sJC0352 | Used as PCR template to make sJC0701 and sJC705 | *NS1* | This work |
| pUC19_ΔNS1_pk2-CcmK1-GFP_ Gm$^{R}$ | sJC0701 | PK2_ccmK1-GFP | *NS1* | This work |

| | | | | |
|---|---|---|---|---|
| pUC19_ΔNS1_pk2-CcmN-GFP_ Gm$^R$ | sJC0705 | PK2_ccmN-GFP | *NS1* | This work |

**Supplementary Table 3. Primers used in this study**

| Name | Description | Sequence (5'-3') | Reference |
|---|---|---|---|
| CHo0005 | Insert amplification of sJC0658 containing Grx1-roGFP2-His for sJC0683 and sJC0691 | GATCCGGGGGTGGTGGGTCTATGGCTCAAGAGTTTGTGAACTG | This work |
| CHo0006 | Insert amplification of sJC0658 containing Grx1-roGFP2-His for sJC0683 and sJC0694 and sJC0691 | GGCTTTGTTAGACAGCCGGATCCTTAGTGATGGTGATGGTGATGAGATC | This work |
| CHo0007 | Vector amplification of sJC0624 containing RbcL and Km$^R$ cassette for sJC0683, sJC0490 containing RbcL and Gm$^R$ cassette for sJC0694, or sJC0490 containing Gm$^R$ cassette for sJC0694 | TAAGGATCCGGCTGTCTAACAAAGCC | This work |
| CHo0008 | Vector amplification of sJC0624 containing RbcL and Km$^R$ cassette for sJC0683 or sJC0490 containing RbcL and Gm$^R$ cassette for sJC0694 | AGACCCACCACCCCCGGATC | This work |
| CHo00010 | Vector amplification of sJC0490 containing Gm$^R$ cassette for sJC0694 | CTAATTTATCCTCGCTTATCAAGCTTAATGTC | This work |
| CHo00011 | Insert amplification of sJC0658 containing Grx1-roGFP2-His for sJC0694 | GCTTGATAAGCGAGGATAAATTAGATGGCTCAAGAGTTTGTGAACTGCAAAAT | This work |
| JCC388 | To test via colony PCR for presence of insert in *glpK* | CAATGGCGAAGGTTTTCTGT | Moore 2020[39] |
| JCC389 | To test via colony PCR for presence of insert in *glpK* | GGGAGATGCTGTAGGCAAGA | Moore 2020[39] |

| | | | |
|---|---|---|---|
| RGo005 | Insert amplification of sJC203 and sJC0201 containing CcmK1-GFP and CcmN-GFP, for sJC0701 and sJC0705, respectively | AATTACTTAAAAACTGAGTAA ATATATATTGCTCGAGTACGG GG | This work |
| RGo006 | Insert amplification of sJC203 and sJC0201 containing CcmK1-GFP and CcmN-GFP, for sJC0701 and sJC0705, respectively | CCAAAGGTCAGAGGAGGATTC ACCTTCGGGAGCG | This work |
| RGo007 | Insert amplification of sJC0352 backbone | CGAGCAATATATATTTACTCA GTTTTTAAGTAATTAGCAGAC AATTCT | This work |
| RGo008 | Insert amplification of sJC0352 backbone | CCCGAAGGTGAATCCTCCTCT GACCTTTGGGC | This work |

**Supplementary Movie 1. Air Movie** Time-lapse imaging of roGFP expressing cells in brightfield and 470 nm excitation/530 nm emission (roGFP2). Cells were grown in air for the full duration of the movie.

**Supplementary Movie 2. Air Redox Movie** Time-lapse ratiometric images with $R_{395/470}$ values ranging from 0 to 0.3. Cells were grown in air for the full duration of the movie. Similar results were observed in two additional independent experiments.

**Supplementary Movie 3. Air to 3% Movie** Time-lapse imaging of roGFP expressing cells in brightfield and 470 nm excitation/530 nm emission (roGFP2). Cells were grown in air for hours 0-12 and in 3% $CO_2$ for hours 12-30.

**Supplementary Movie 4. Air to 3% $CO_2$ Redox Movie** Time-lapse ratiometric images with $R_{395/470}$ values ranging from 0 to 0.3. Cells were grown in air for hours 0-12 and in 3% $CO_2$ for hours 12-30. Similar results were observed in two additional independent experiments.

**Supplementary Movie 5. 3% $CO_2$ to Air Movie** Time-lapse imaging of roGFP expressing cells in brightfield and 470 nm excitation/530 nm emission (roGFP2). Cells were grown in 3% $CO_2$ for hours 0-12 and in air for hours 12-30.

**Supplementary Movie 6. 3% $CO_2$ to Air Redox Movie** Time-lapse ratiometric images with $R_{395/470}$ values ranging from 0 to 0.3. Cells were grown in 3% $CO_2$ for hours 0-12 and in air for hours 12-30. Similar results were observed in two additional independent experiments.

**Supplementary Movie 7. Tomogram of RbcL-Grx1-roGFP2 grown in Air.**

**Supplementary Movie 8. Tomogram of RbcL-Grx1-roGFP2 grown in 3%$CO_2$.**

**Supplementary Movie 9. Tilt Series of WT grown in Air.**

**Supplementary Movie 10. Tilt Series of *ΔccmO* RbcL-Grx1-roGFP2 grown in 3%$CO_2$.**
